# Study on the Behavior of Electrochemical Extraction of Cobalt from Spent Lithium Cobalt Oxide Cathode Materials

**DOI:** 10.3390/ma14206110

**Published:** 2021-10-15

**Authors:** Hui Li, Haotian Li, Chenxiao Li, Jinglong Liang, Hongyan Yan, Zhengzhen Xu

**Affiliations:** College of Metallurgy and Energy, North China University of Science and Technology, Tangshan 063210, China; lh@ncst.edu.cn (H.L.); li986496033@163.com (H.L.); lichenxiao@ncst.edu.cn (C.L.); yanhy@ncst.edu.cn (H.Y.); xzz15131511868@163.com (Z.X.)

**Keywords:** NaCl-CaCl_2_-LiCoO_2_, Co, electrochemical test, electrolysis experiments

## Abstract

The molten salt electrochemical method was used to reduce the Co in spent LiCoO_2_. The reduction mechanism of Co (III) in LiCoO_2_ was analyzed by cyclic voltammetry, square wave voltammetry, and open circuit potential. The reduction process of Co (III) on Fe electrode was studied in NaCl-CaCl_2_-LiCoO_2_ molten salt system at 750 °C. The results show that the reduction process of Co (III) is a two-step reduction: Co (III) → Co (II) → Co (0) and they are all quasi-reversible processes controlled by diffusion. Phase analysis (XRD) shows that Li^+^ and Cl^2−^ in the molten salt form LiCl electrolysis experiments with different voltages were carried out, which proved the stepwise reduction of Co in LiCoO_2_.

## 1. Introduction

Lithium-ion batteries have many excellent electrochemical properties, such as a large number of charge and discharge times and a long cycle life. In addition, lithium-ion batteries have no memory effect. As the number of recharges increases, their power storage will not decrease. Lithium-ion batteries are widely used in small and medium-sized battery industries, such as mobile phones, portable notebooks, and electric vehicles [1,2,3]. Therefore, people’s demand for and purchase of lithium-ion batteries has increased year by year, which has led to a large number of spent lithium-ion batteries (LIBs) in the country. After three to five years of cycles, lithium-ion batteries will eventually be scrapped. By 2020, the scrap volume reached about 400 million tons [4]. Spent lithium-ion batteries are difficult to degrade and have a certain toxicity. The random disposal of used lithium-ion batteries will seriously affect the natural ecology and ultimately endanger human health. In addition, spent LiCoO_2_-based lithium-ion batteries contain very high-grade rare metals lithium and cobalt [5,6]. Therefore, the recovery of high-grade metals from waste lithium-ion batteries will have a certain improvement in the pressure on the environment and resources [7,8].

Traditional methods for metal recovery from used lithium-ion batteries include pyrometallurgy, hydrometallurgy, and biometallurgy. Due to the harsh microbial culture conditions, the leached heavy metals are toxic to microorganisms, and the slow kinetics of the metallurgical process, biometallurgical methods have great limitations in commercial applications [9,10,11]. Pyrometallurgy is accompanied by shortcomings such as high energy consumption and strict requirements for processing equipment [12]. Hydrometallurgy uses acids and extractants to recover metals. The organic solvents used are complex and cumbersome. Excessive acid will also produce by-products such as Cl_2_, SO_x_, and NO_x_. This causes great pollution to the environment and does not conform to the concept of environmental protection [13,14,15,16]. Ma et al. [17] have invented a method of low-temperature chlorination pyrolysis of waste lithium-ion batteries in order to reduce energy consumption and not use acid-base agents. This method recovers valuable metals into the leaching solution in one step at 300 °C, and the leaching rate is as high as 99%. Zhang et al. [18] used pollution-free grape seeds and malic acid as the leaching night successfully recovered 92% Co and 99% Li from spent LIBs. Therefore, finding suitable energy saving methods has attracted widespread attention.

In recent years, the electrometallurgical technology in line with the concept of green environmental protection has received extensive attention. Chang Wei et al. [19] put the spent lithium-ion battery as the cathode in the electrolyte with anhydrous sodium sulfate as the electrolyte, and the current density, the influence of different concentrations of sulfuric acid and citric acid on the leaching of cobalt and aluminum were investigated. Finally, the current density was 15.6 mA/cm^2^. The leaching rate of cobalt and aluminum in waste lithium-ion batteries with a concentration of 40 g/L sulfuric acid or a concentration of 36 g/L citric acid reached 90.8% and 7.9%. In addition to the aqueous solution electrolysis of waste lithium-ion batteries, Yin Huayi et al. [20] successfully prepared metal Li and Co by electrolysis of LiCoO_2_ at 1023 K and molten salt of Na_2_CO_3_-K_2_CO_3_ for waste LiCoO_2_-based lithium-ion batteries. The metal recovery rate was 85% and 99%, respectively. The electrolyzed CoO and Li_2_CO_3_ combine to form LiCoO_2_, so that LiCoO_2_ can be recycled. This simple, comprehensive and green process provide a good idea for the recovery of metallic Co from waste LiCoO_2_-based batteries.

When we extract metallic Co from LiCoO_2_, first we need to understand the reduction mechanism of LiCoO_2_ to Co. In this paper, molten salt electrochemical method is adopted at 750 °C and NaCl-CaCl_2_-LiCoO_2_ molten salt system are selected, combined with electrochemical test methods to analyze the Co (III) reduction behavior of LiCoO_2_ in molten salt. The electrolysis experiment of LiCoO_2_ with different voltages confirmed the process of LiCoO_2_ reduction to Co.

## 2. Experimental Method

The raw materials are LiCoO_2_ (purity ≥ 99.95), NaCl (purity ≥ 99.5), and CaCl_2_ (purity ≥ 99.5) purchased from Sinopharm Group. The corundum crucible (Zhongkehuaxueci, Tangshan, China) is used, and the main component is Al_2_O_3_ (purity ≥ 99.95). JJ124BC electronic balance (Shuangjie, Changshu, China) is used for weighing. DZF-6020 vacuum (Boxun, Shanghai, China) drying oven is used for drying, and 3KL10·BYL resistance furnace (Yunjie, Baotou, China) is used for electrochemical test and electrolysis experiment. The electrochemical test uses the CHI660E electrochemical workstation (Chenhua, Shanghai, China) and the Noran7 X-ray diffractometer (Rigaku, Tokyo, Japan) is used for phase analysis of the raw materials and products.

The electrochemical test was carried out using a three-electrode system, iron wire (purity ≥ 99.99%, Φ1 mm) chosen as the working electrode (WE) for investigating electrochemical behaviors of Co (III). The counter electrode (CE) was a smooth graphite sheet (purity ≥ 99.99%, 10 mm × 5 mm × 90 mm) polished with 1500#–2000#. The platinum wire (purity ≥ 99.999%) in the alumina tube (Φ4 mm) was used as the reference electrode (RE). First, the NaCl and CaCl_2_ were placed in a vacuum drying (Boxun, Shanghai, China) oven at 573 K for 5 h to fully remove the moisture in NaCl and CaCl_2_. Then 200 g of molten salt was taken with *n*NaCl: *n*CaCl_2_ = 1:1 to crush and mix, the mixed salt was then put in the corundum crucible and moved to the drying box for drying. When using mixed salt, it is important to take out the crucible containing the mixed salt and put it into the resistance heating furnace (Yunjie, Baotou, China). The temperature was raised to 750 °C at 5 °C/min, argon gas passed through the whole process for protection. When the temperature rose to 750 °C, the temperature was kept constant for 1 h to fully dissolve the medicine, then the three electrodes were put into the upper part of the furnace tube for preheating for 10 min to prevent the three electrodes from being affected by extreme heat, and then insert the electrodes in the molten salt to perform the blank salt system Electrochemical detection. After the measurement, the electrode is taken out, 4 g of LiCoO_2_ powder is added to the molten salt, and the temperature is kept constant for 4 h to make the LiCoO_2_ saturated in the NaCl-CaCl_2_-LiCoO_2_ molten salt system, and the three electrodes are placed again for electrochemical detection.

The electrolysis experiment was carried out with a two-electrode system and 1 g of LiCoO_2_ powders were compressed into a sheet after 4 MPa 2 min. (Diameter of about 15 mm, the thickness of about 2 mm). Then it was placed in a resistance furnace and sintered at a temperature of 5 °C/min to 700 °C for 10 h to improve the mechanical strength of the sheet. The sintered LiCoO_2_ pellets were then wrapped in stainless steel mesh cloth and fixed on a stainless-steel rod as a cathode, and a high-purity graphite flake (purity ≥ 99.99%, 10 mm × 5 mm × 90 mm) was fixed on another stainless-steel rod as an anode. The constant cell voltage electrolysis voltage range was 0.5–1.5 V. After the electrolysis, the cathode product was cooled with deionized water and cleaned by ultrasonic cleaning, and then placed in a drying box to dry for 12 h. Then the product was subjected to XRD detection.

## 3. Experimental Results and Discussion

### 3.1. Cyclic Voltammetry

#### 3.1.1. Cyclic Voltammetry of NaCl-CaCl_2_-LiCoO_2_

**Figure 1:**
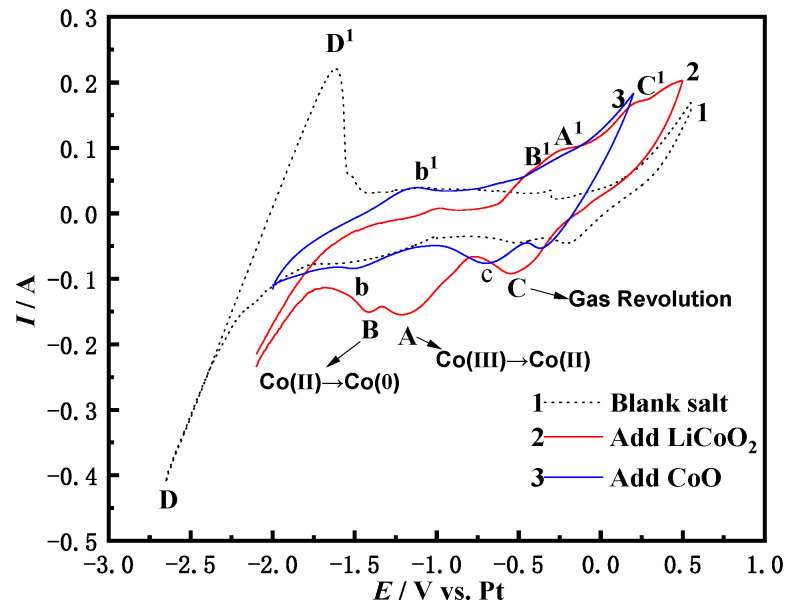
Cyclic voltammetry comparison of pure salt in NaCl-CaCl_2_-LiCoO_2_ molten salt and addition of oxide (0.2 V/S; WE: Fe electrode).

The dotted line in Figure 1 is the NaCl-CaCl_2_ blank experiment. From the blank experiment, the reduction and oxidation peak of the leftmost DD^1^ can be observed. This may be the peak corresponding to Ca^2+^ and Ca or the corresponding peak of Na^+^ and Na [21], because the thermodynamic calculation software HSC Chemistry 6.0 is adopted by Formula (1). It is calculated that the theoretical decomposition voltages of NaCl and CaCl_2_ at 1023 K are −3.238 and −3.325 V, respectively, and there is not much difference between the two, which makes it difficult to distinguish the positions of peaks. When the potential range is between −2.0 and −0.5 V, there is no corresponding peak, so there is no oxidation-reduction reaction in the molten salt system at this time. This range can be used as the potential window after LiCoO_2_ is added to the NaCl-CaCl_2_ molten salt. The red curve 2 is the NaCl-CaCl_2_ added LiCoO_2_. It is shows three pairs of redox peaks, namely C (−0.5 V) and C^1^, A (−1.189 V) and A^1^, B (−1.431 V) and B^1^. During the negative scanning of the potential, the current increased and a large number of active electrons accumulated on the surface of the cathode. The electrode deviated from the equilibrium potential and exhibited a polarization phenomenon, attracting Li^+^ and Co^3+^ in the molten salt to the cathode, and the current gradually increased, which is the process of forming an electric double layer with the molten salt phase. When the potential was −0.5 V, the first reduction peak C (−0.5 V) appeared and the corresponding oxidation peak was C^1^, which is related to the process of the gas revolution reaction [22]. As the negative scanning of the potential, the potential reached the initial potential formed by peak A, showing that a large amount of Co^3+^ in the molten salt diffused to the surface of the electrode. When the potential reached the energy required for Co^3+^ to participate in the reduction reaction, the process of Co^3+^ causing electrons to form Co^2+^ occurred on the surface of the electrode. Due to the high concentration of Co^3+^ near the cathode, at this time, the reaction rate was fast and the current increased. However, the generated Co^2+^ occupied the active sites on the electrode surface, which prevented the subsequent Co^3+^ diffused from the molten salt from being reduced to Co^2+^ on the electrode, resulting in a decrease in the reaction rate and current, which eventually formed A (−1.189 V) of the reduction peak current [20]. When the negative scanning potential continued to increase to the overpotential of the reduction of Co^2+^ on the cathode surface, the Co^2+^ on the electrode surface obtained electrons to form Co, and the active sites on the electrode surface were converted to being occupied by Co. The new redox reaction made the reaction rate speed up again and the current continued to increase The Co^2+^ on the electrode surface gradually decreased due to the formation of Co. At the same time, the Co^3+^ in the molten salt could not be quickly reduced to Co^2+^ because the active sites were occupied by Co. The reaction to form Co was inhibited. The cumulative result of the two meant that the current was unable to continue to increase. Finally, the third reduction peak B (−1.431 V) appeared. Therefore, LiCoO_2_ in the molten salt has undergone a gradual reduction process of Co (III) → Co (II) → Co (0), which is initially certified from the blue curve 3. The addition of CoO to the molten salt has some deviation compared with the addition of LiCoO_2_. The Co in CoO is + 2 valence, except for the gas revolution reaction peak c, only one reduction peak b appears in the curve. Therefore, it can be seen that the reduction process of Co^2+^ on the Fe electrode in the NaCl-CaCl_2_ molten salt system is Co (II) → Co (0). The number of electrons transferred by the reaction is calculated in Section 3.2.

(1) $$\Delta G^{\Theta }=-nFE^{\Theta }$$

#### 3.1.2. Cyclic Voltammetry of NaCl-CaCl_2_-LiCoO_2_ at Different Scan Rates

**Figure 2:**
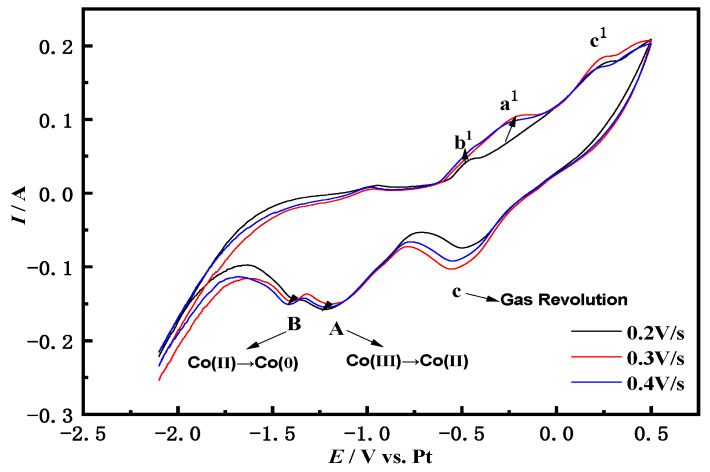
NaCl-CaCl_2_-LiCoO_2_ molten salt system multi-cycle cyclic voltammetry (0.2–0.4 V/s; Fe electrode).

Figure 2 shows the cyclic voltammetry curves of the Fe electrode in the NaCl-CaCl_2_-LiCoO_2_ system measured at different sweep speeds at 750 °C. As the scanning speed increases, the potential change per unit time is greater, the time required for electric double layer charging becomes shorter, the electrochemical reaction speed of the electrode surface is accelerated, and the current value changes more steeply, making the peak current larger.

The two pairs of redox peaks Aa^1^ and Bb^1^ show a slight peak shift to the negative direction for a cathodic reaction and toward the positive direction for an anodic reaction, with an increase in the scan rates revealing a quasi-reversible or irreversible. The values of the redox peak currents of peaks Aa^1^ and Bb^1^ at different sweep rates can be further judged by Formula (2) to determine whether the A and B reaction steps are quasi-reversible. The Equations are as follows:

(2) $$\left|I_{pc}/I_{pa}\right|=1$$

**Figure 3:**
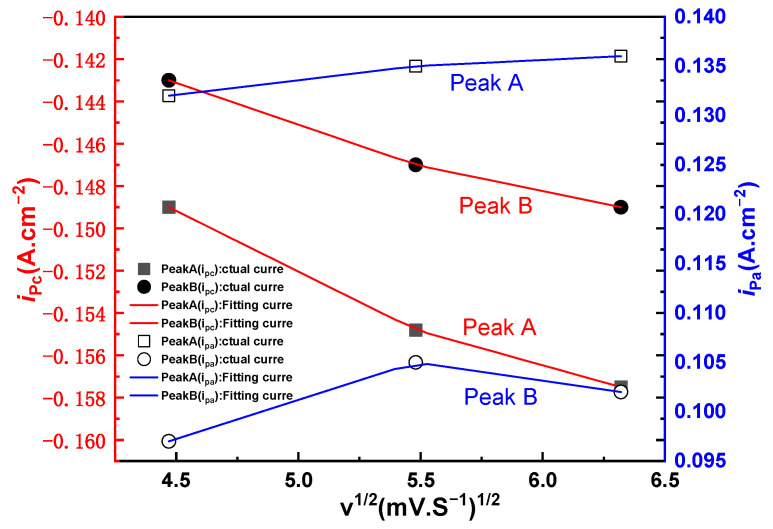
Relationship between *i_pc_* and *v*^1/2^ of Fe electrode in NaCl-CaCl_2_-LiCO_2_ molten salt system.

It is calculated that the peak current ratio of redox peaks Aa^1^ and Bb^1^ is approximately equal to 1. The relationship between *i_pc_* and *v*^1/2^ of the scan rate is further determined whether it is controlled by diffusion. The *i_pc_* and *v*^1/2^ of the reduction peaks A and B are drawn by extracting the data from the cyclic voltammetry curves of different scanning speeds, and linear fitting is performed to obtain the following Figure 3. It can be seen from the Figure 3 that the reduction peak currents of A and B have a linear relationship with *v*^1/2^. Based on the above three criteria, it is concluded that the two reactions A and B are quasi-reversible processes and are controlled by diffusion [23].

### 3.2. Square Wave Voltammetry

**Figure 4:**
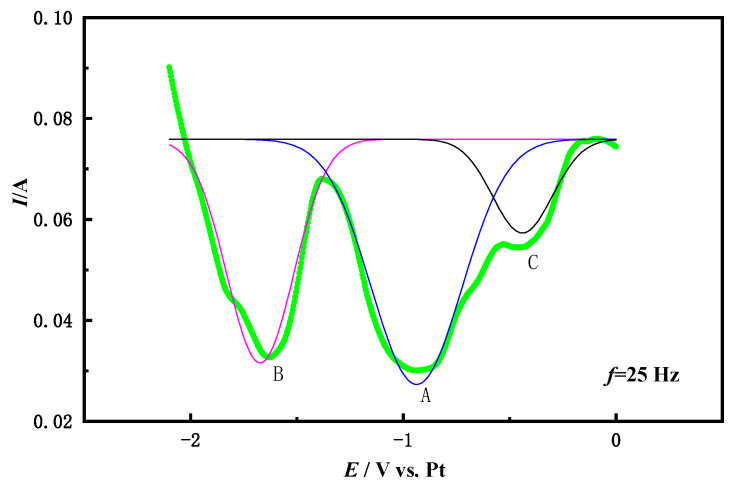
Square wave voltammetry of NaCl-CaCl_2_-LiCoO molten salt system (25 Hz; Fe electrode).

The number of electrons transferred by Co^3+^ on the Fe electrode was studied by the square wave voltammetry. Figure 4 shows the square wave voltammetry curve on the Fe electrode in the NaCl-CaCl_2_-LiCoO_2_ molten salt with a frequency of 25 Hz and peak fitting. Two strong reduction peaks still appear on the curve: A is −0.916 V and B is −1.6 V. Compared with the above cyclic voltammetry curve, it can be seen that the A and B peaks correspond to the two-step reduction of Co. According to the half-width of the quasi-reversible coincidence (*W*_1/2_) Formula (3) [24] calculates the number of transferred electrons (*n*) of Co^3+^ in LiCoO_2_.

(3) $$W_{1/2}=3.52\frac{RT}{nF}$$

In the formula: *R*—molar gas constant (8.3145 J/mol/K); *T*—temperature (K); *n*—electron transfer number; *F*—Faraday’s constant (96,485 C/mol).

According to the square wave voltammogram, the *W*_1/2_ value corresponding to peak A is about 0.18 V, and the *W*_1/2_ value corresponding to peak B is about 0.22 V. The number of electrons transferred by the reduction peak of A is calculated by the Formula (3) *n* = 0.9, and the number of electrons transferred by the reduction peak of B is *n* = 1.5. Therefore, it is determined that Co (III) is a two-step reduction process, namely:

Co (III) + e^−^ = Co (II) (4)

Co (II) + 2e^−^ = Co (0) (5)

The above analysis shows that the reduction process of Co (III) on the Fe electrode is a two-step process of obtaining electrons, Co (III) → Co (II) → Co (0), which is consistent with the conclusion of the cyclic voltammetry curve analysis.

### 3.3. Open Circuit Chronopotential

**Figure 5:**
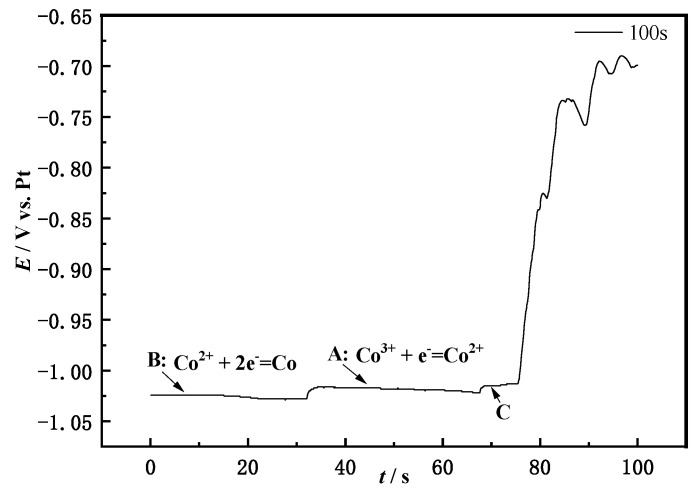
Open circuit chronopotential of NaCl-CaCl_2_-LiCoO_2_ molten salt system (100 s; Fe electrode).

The reduction step of LiCoO_2_ is judged from the number of platforms where the open circuit potential appears. Figure 5 shows the open circuit in the NaCl-CaCl_2_-LiCoO_2_ molten salt system. First apply −1.5 V voltage to the working electrode for polarization for the 60 s, so that the working electrode has higher energy to convert LiCoO_2_ into metal Co, and then perform an open circuit potential test. It can be seen from the Figure 5 that there are three obvious platforms. Platform B first appears on the surface of the working electrode. Its potential is −1.025 V, which corresponds to the peak B in the cyclic voltammetry curve. The reaction here is Co (0) → Co (II). As time goes by, the concentration of Co^2+^ keeps increasing, and the electrode potential keeps decreasing. When the potential decreases to the potential for Co^2+^ oxidation reaction, the second plateau A is reached at this time, and its potential is −1.01 V, which corresponds to the potential of peak A in the cycle voltammetry. The reaction occurring is Co (II) → Co (III).

### 3.4. Cyclic Voltammetry of Li_2_O in NaCl-CaCl_2_

**Figure 6:**
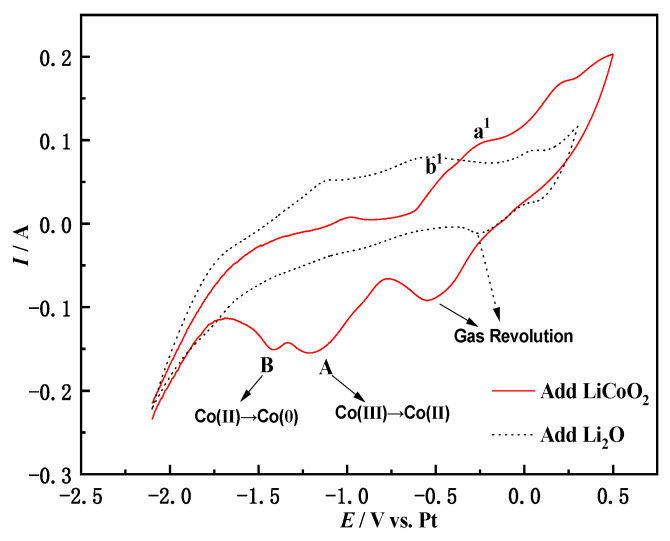
Comparison of cyclic voltammetry curves after addition of Li_2_O with LiCoO_2_ (CaCl_2_-NaCl system 0.2 V/S Fe electrode).

**Figure 7:**
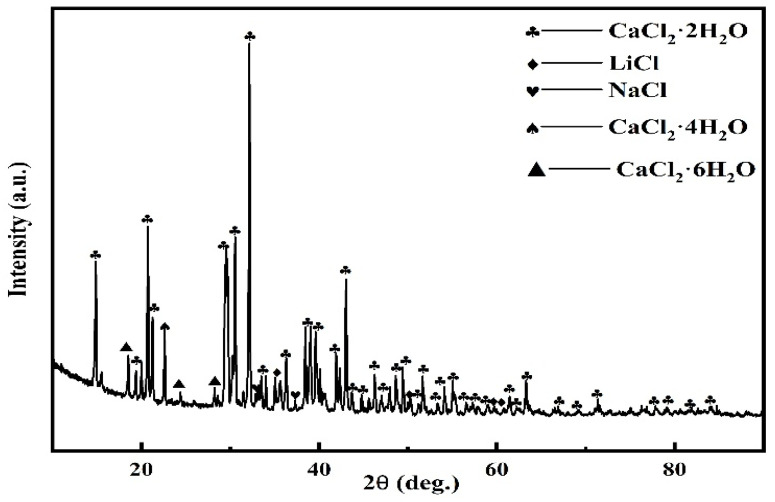
XRD detection of molten salt near the cathode after electrolysis.

The two-step reduction of Co (III) → Co (II) → Co (0) in LiCoO_2_ has been verified from multiple angles such as cyclic voltammetry, square wave voltammetry and open-circuit chronopotential. However, the Li^+^ fate problem is not obtained by the above method. Figure 6 shows the comparison of cyclic voltammetry between adding Li_2_O and LiCoO_2_ in molten salt. It can be seen from Figure 6 that the cyclic voltammetry curve of adding Li_2_O in molten salt has no redox peak in the potential window. This is because the theoretical decomposition voltage of Li is more negative than that of Co and Fe. Equation (6) combined with Equation (1) calculates the decomposition voltage of Li_2_O as −2.34 V, which means that its reduction occurs after Fe and Co. Therefore, during the electrochemical scanning process, the redox peak of Li did not appear. According to Equations (7) and (8) and Figure 7, XRD analysis of the molten salt near the cathode after electrolysis, it can be concluded that Li^+^ will eventually dissolve spontaneously into the molten salt to form LiCl.

4 Li + O_2_ = 2 Li_2_O ΔG^Θ^ = −923.7115 kJ/mol (6)

Li_2_O + 2 NaCl = 2 LiCl + Na_2_O ΔG^Θ^ = 164.5 kJ/mol (7)

Li_2_O + CaCl_2_ = 2 LiCl + CaO ΔG^Θ^ = −80.7 kJ/mol (8)

### 3.5. Preparation of Co by Electrolysis of LiCoO_2_ at Constant Cell Pressure

#### 3.5.1. Immersion Test of LiCoO_2_ in NaCl-CaCl_2_ Molten Salt

**Figure 8:**
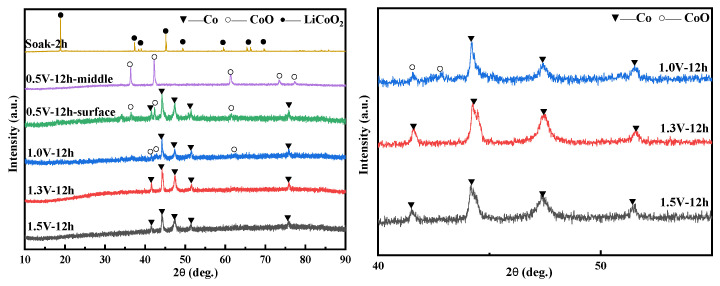
XRD of products electrolyzed at different voltages for 12 h.

The sintered 1 g LiCoO_2_ sheet was made into a cathode, and soaked in NaCl-CaCl_2_ molten salt at 750 °C for 2 h to complete the immersion experiment, and explore whether it would have a chemical reaction in the molten salt; the XRD of the immersion experiment is shown in Figure 8, and it was found LiCoO_2_ did not react after being immersed for 2 h. It can be seen that LiCoO_2_ is physically dissolved in molten salt. After eliminating the interference of molten salt on the raw material LiCoO_2_, electrolysis experiments were carried out under different voltage conditions.

#### 3.5.2. The Influence of Voltage Conditions on Electrolysis

**Figure 9:**
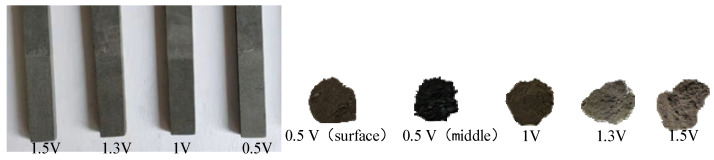
Images of anode (**left** is graphite sheet) and cathode (**right** is products after electrolysis) products of graphite rods electrolyzed at different voltages for 12 h.

It can be seen in Figure 8 that CoO and metallic Co were formed on the surface of the cathode sheet at 0.5 V and 12 h, while the internal products were all CoO. Figure 9 more intuitively shows that the surface of the cathode of the 0.5 V product is gray in Co, while the inside of the product shows a black color containing a mixture of CoO and Co. This reduction process conforms to the 3PIs reaction model. The electrolysis reaction slowly extends from the three contact points of the current collector/LiCoO_2_ sheet/molten salt to the entire cathode surface. The internal O^2−^ removal path becomes longer, and the reaction speed is slower than the cathode surface. Therefore, 0.5 V, 12 h shows Co production on the outside and CoO on the inside. This also confirms the two reduction processes of LiCoO_2_. In Figure 9, the anode graphite sheet gradually becomes rough with the increase in voltage, indicating that O^2−^ diffuses faster in the reaction, and more O^2−^ diffuses to the anode to discharge. When the voltage rises to 1.3 V, all LiCoO_2_ is reduced to Co. In addition, when the voltage is increased to 1.5 V, the characteristic peak width of the electrolytic product Co is smaller, indicating that Co has undergone a longer crystal growth stage during the reaction time, and its crystal size is larger than that in the 1.3 V product.

## 4. Conclusions

(1) When the potential is within −2.6 V~0.5 V, two pairs of redox peaks appear, indicating that the reduction of Co (III) is a two-step process, Co (III) → Co (II) → Co (0). In the first step, the Co (III) in the molten salt is reduced to Co (II) by an electron on the surface of the Fe electrode, and the second step is that Co (II) on the active site of the Fe electrode is reduced to Co (0) by two electrons. The reduction process of LiCoO_2_ is a quasi-reversible reaction controlled by diffusion. Li^+^ forms LiCl with Cl^−^ in molten salt during electrochemical reaction.
(2) Electrolysis experiments with different voltages of 0.5–1.5 V have confirmed the two-part reduction process of LiCoO_2_. The reduction reaction first proceeds on the three-phase boundary of the current collector/LiCoO_2_ sheet/molten salt contact, and then extends to the entire cathode surface. The inside of the LiCoO_2_ sheet reaction speed is slower than the surface. The inside of the LiCoO_2_ sheet is all CoO, which is the first reduction product, while CoO and Co are present on the outside at 0.5 V,12 h. When the voltage reaches 1.3 V, the LiCoO_2_ sheet is reduced to Co.

## Data Availability

Data sharing is not applicable to this article.
